# Alcohol acyltransferases for the biosynthesis of esters

**DOI:** 10.1186/s13068-023-02343-x

**Published:** 2023-06-01

**Authors:** Gaofei Liu, Lei Huang, Jiazhang Lian

**Affiliations:** 1 Key Laboratory of Biomass Chemical Engineering of Ministry of Education, College of Chemical and Biological Engineering, Zhejiang University, Hangzhou, 310027 China; 2 ZJU-Hangzhou Global Scientific and Technological Innovation Center, Zhejiang University, Hangzhou, 311215 China; 3 Zhejiang Key Laboratory of Smart Biomaterials, Zhejiang University, Hangzhou, 310027 China

**Keywords:** Ester biosynthesis, Alcohol acyltransferase, Protein engineering, Metabolic engineering, Synthetic biology

## Abstract

Esters are widely used in food, energy, spices, chemical industry, etc., becoming an indispensable part of life. However, their production heavily relies on the fossil energy industry, which presents significant challenges associated with energy shortages and environmental pollution. Consequently, there is an urgent need to identify alternative green methods for ester production. One promising solution is biosynthesis, which offers sustainable and environmentally friendly processes. In ester biosynthesis, alcohol acyltransferases (AATs) catalyze the condensation of acyl-CoAs and alcohols to form esters, enabling the biosynthesis of nearly 100 different kinds of esters, such as ethyl acetate, hexyl acetate, ethyl crotonate, isoamyl acetate, and butyl butyrate. However, low catalytic efficiency and low selectivity of AATs represent the major bottlenecks for the biosynthesis of certain specific esters, which should be addressed with protein molecular engineering approaches before practical biotechnological applications. This review provides an overview of AAT enzymes, including their sequences, structures, active sites, catalytic mechanisms, and metabolic engineering applications. Furthermore, considering the critical role of AATs in determining the final ester products, the current research progresses of AAT modification using protein molecular engineering are also discussed. This review summarized the major challenges and prospects of AAT enzymes in ester biosynthesis.

## Introduction

Esters are widely used in agriculture, food and cosmetics, as well as serving as solvents and biofuels [1–7]. Insecticides like carbamates and pyrethroids have been used worldwide [8, 9]. Volatile esters play a crucial role in the flavor of alcoholic beverages [10], such as isobutyl acetate with a fruit-like aroma [11] and Chinese liquor with most abundant and vital aromatic esters [12, 13]. Benzyl acetate, linalyl acetate, geranyl acetate, and citronellyl formate are the main substances in plant essential oils and spices [14, 15]. Esters can be regarded as eco-friendly solvents, due to their excellent solubility and biodegradability [16], with ethyl acetate as the most commonly used industrial solvent and ethyl lactate as the new generation of green solvents [17, 18]. Fatty acid alkyl esters form an essential component of biodiesel [19] with butyl butyrate and ethyl octanoate as potential alternative jet fuels as well as ethyl valerate, butyl butyrate, butyl valerate, and pentyl valerate as good fuel additives for gasoline [3, 20–22].

The conventional method for industrial production of esters involves Fischer–Speier esterification [23, 24]. However, this process is associated with high energy consumption and the generation of hazardous waste, as demonstrated by the synthesis of ethyl acetate from acetic acid and ethanol by concentrated sulfuric acid at 200–250 ℃ [25]. In view of the current energy shortage and environmental protection-related challenges, there is an urgent need to identify alternative green methods for ester production [4]. In the past two decades, metabolic engineering has demonstrated its potential to convert renewable resources into a range of chemical products with high yield and selectivity [26–29], offering powerful biotechnology support for the green production of esters. Microbial conversion systems have already received much attention for sustainable bulk chemical production [30–32] and will be key in developing efficient ester-producing bioprocesses.

**Fig. 1:**
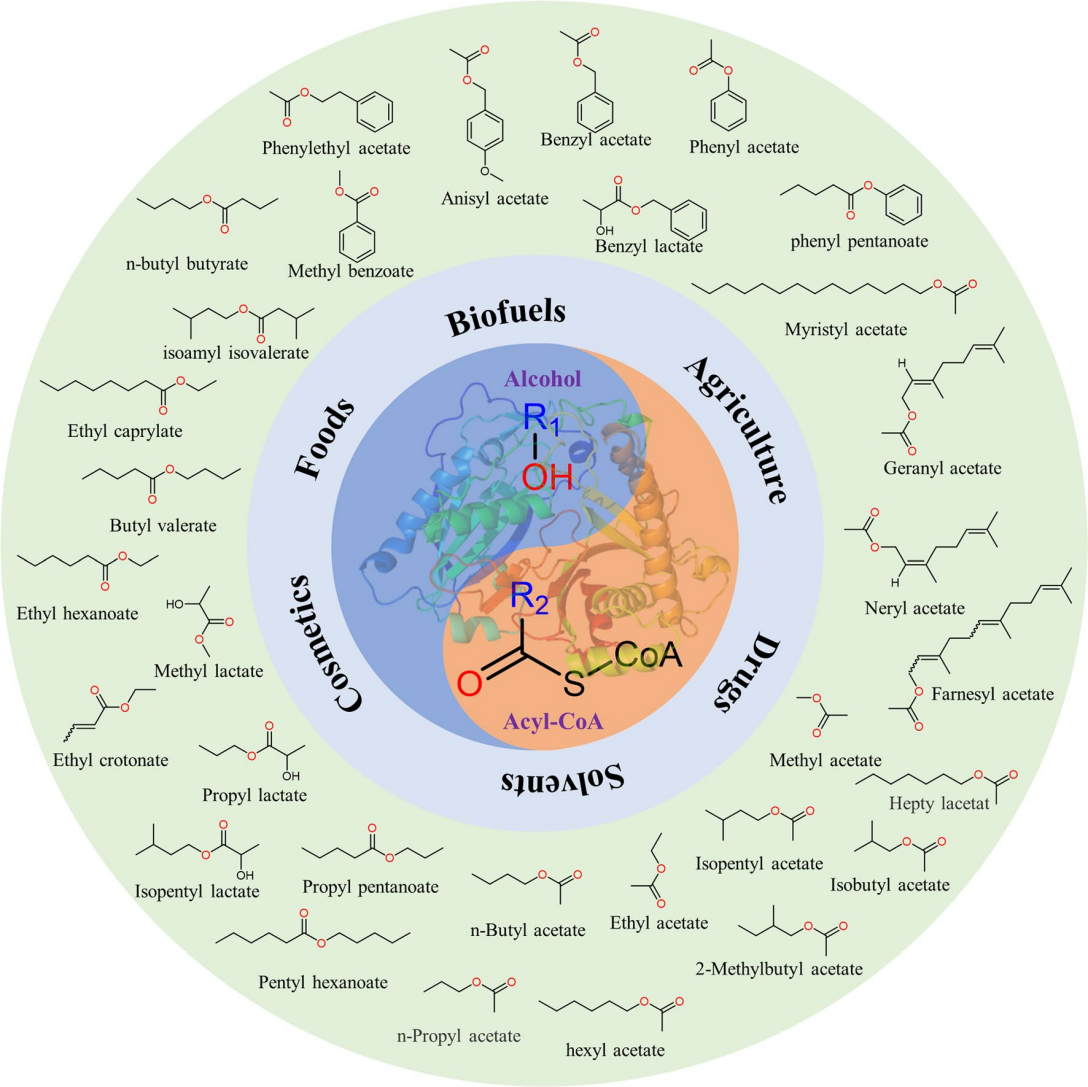
Ester compounds synthesized by AATs

To date, four types of enzymes involved in the formation of esters have been identified: esterase, hemiacetal dehydrogenation (HADHs), Baeyer–Villiger monooxygenase (BVMOs), and alcohol acetyltransferases (AATs). Esterase (e.g., lipase) [33, 34] can catalyze the formation of esters by reacting organic acids with organic alcohols in a non-aqueous phase [35]. This field has been widely studied and previously reviewed [36]. HADHs mainly exist in methylotrophic yeast, such as *Pichia pastoris* [37]. When high concentrations of formaldehyde and acetaldehyde accumulate, they are converted by HADHs to form esters, leading to aldehyde detoxification. This process requires NAD(P)^+^ as a hydrogen acceptor [38]. BVMOs catalyze the conversion of aldehydes and ketones to esters with the insertion of oxygen between C–C bonds. This group of enzymes has been extensively studied and shown to play an important role in the biosynthesis of various esters [39–41]. AATs are a large and diverse group of enzymes that catalyze the condensation of acyl-CoA and alcohol to form the corresponding esters [42–48]. Due to their ability to accept different types of acyl-CoAs and alcohols, AATs can synthesize many natural and artificially designed esters (Fig. 1), making them the most promising group of enzymes in esters biosynthesis [49, 50].

This review article provides an overview of the current research progress on AATs. Specifically, we focus on the sequences, structures, active sites, and catalytic mechanisms of AATs. We also introduce the improvement of the enzymatic performance of AATs (e.g., catalytic activity, substrate selectivity, thermal stability, and soluble expression) via protein molecular engineering approaches. Furthermore, we discuss the practical applications of AATs in the biosynthesis of esters. Finally, we summarize the main challenges of AATs as key enzymes in the biosynthesis of ester compounds.

## Structure and sequence features of AATs

**Fig. 2:**
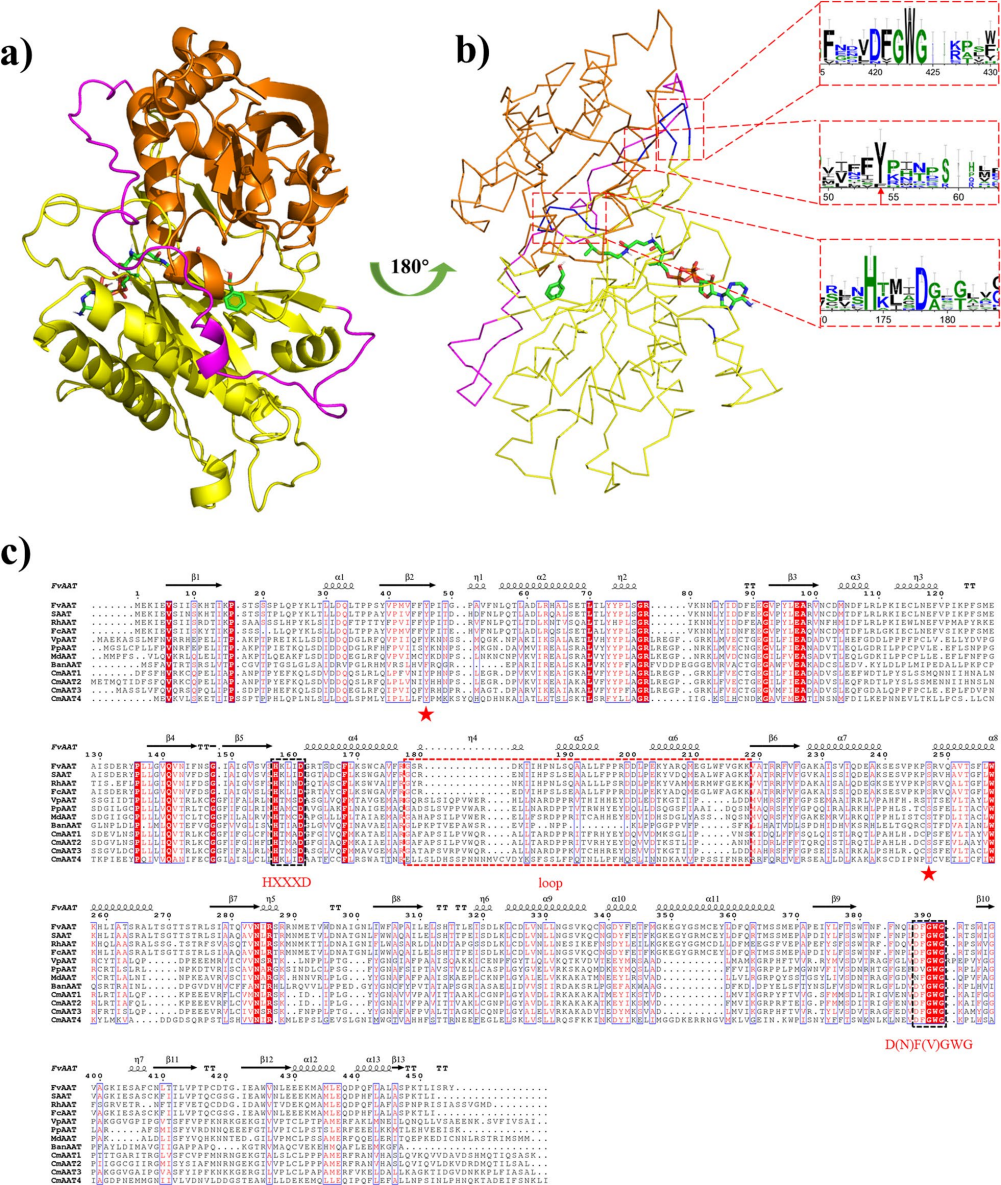
Structure and sequence features of AATs. **a** Structure diagram of FvAAT (AAT from *Fragaria vesca* [119]). 3D homology modeling of FvAAT was conducted using RoseTTAFold with 2BGH (PDB accession code) as the template. FvAAT consists of two domains (yellow and orange), joined by a crossover loop (magenta). Benzyl alcohol and acetyl-CoA (shown as sticks) were chosen as the representative substrates for molecular docking with FvAAT. The overall fold is represented by a cartoon and ribbon diagram. All structural figures in this article were prepared using Pymol software. **b** Sequence conservation analysis diagram of AATs using Weblogo (http://weblogo.berkeley.edu/logo.cgi). **c** Multiple sequence alignments of AATs. Comparison of amino acid sequences was aligned using the ClustalW2 server. The residues marked with red asterisk are the important amino acids identified. The important motifs in the black dotted box and a loop connecting two domains are in the red dotted box. FvAAT (NP_001295454); SAAT (*Strawberry*, AF193789_1); RhAAT (*Rosa hybrid cultiva*r, AAW31948); FcAAT (*Fragaria chiloensis*, ACT82247); VpAAT (ACT82248); PpAAT (*Prunus persica*, XP_007209131); MdAAT (*Malus domestica*, AAT2_MALDO); BanAAT (*Musa acuminata*, CAG1859434); CmAAT1 (KAA0066373); CmAAT2 (AF468022_1); CmAAT3 (NP_001315395); CmAAT4 (NP_001315389). FvAAT has 463 amino acids with a molecular weight of 51.4 kDa, which has two conserved regions typical of the BADH superfamily HXXXD and DFGWG (amino acids 381–385 in FvAAT) near the C-terminus [60]

AATs are part of the BAHD superfamily, named after the initial letters of four enzymes: benzyl alcohol *O*-acetyltransferase (BEAT) [51, 52], anthocyanin *O*-hydroxycinnamoyltransferase (AHCT) [53], anthranilate hydroxycinnamoyl/benzoyltransferase (HCBT) [54], and deacetylvindoline 4-*O*-acetyltransferase (DAT) [55]. In higher plants, the BAHD acyltransferase family is responsible for the biosynthesis of various natural products, including esters. Although the BAHD superfamily demonstrates high sequence divergence (less than 30% of identity), the subunit fold is remarkably conserved, with similar compositions and distributions of α-helices and β-sheets. These proteins consist of two domains (yellow and orange) of comparable sizes, joined by a crossover loop (magenta) (Fig. 2a).

Vinorine synthase, an acetyltransferase from *Rauvolfia serpentina*, is the first member from the BAHD superfamily to have its crystal structure (PDB ID: 2BGH) solved at 2.6 Å resolution [56]. As shown in Fig. 2b, the HXXXD motif, highly conserved sequences located in the middle of proteins from higher plants and yeasts, is essential for enzyme function [57]. The His residue is directly involved in catalysis, while the Asp residue plays a structural role in maintaining the channel structure. For vinorine synthase, the substitution of the histidine and aspartic residues from this motif results in a loss of enzyme activity [58]. Similar results have been observed in other members of the BAHD superfamily, including AAT from *Vasconcellea pubescens* (VpAAT), where the substitution of H166 and D170 in the HXXXD motif resulted in a loss of enzyme activity [59]. These findings suggest that the HXXXD motif is involved in the transfer of the acyl group from acyl-CoAs to the alcohol substrates.

The DFGWG sequence is another highly conserved motif of the BAHD superfamily in higher plants, locates near the C-terminus of proteins (Fig. 2b) [60]. Structural analysis suggests that this motif is unlikely to play a direct role in substrate binding or catalysis, as it is remote from both the active site and solvent channel [56, 61], which appears to be involved in maintaining structural integrity. Based on the crystal structure of vinorine synthase [56], residue D362 stabilizes the turn of *β* sheet 11 to *β* sheet 12 by forming hydrogen bonds with the amide group of W365 and G366 (G385 in VpAAT) of the DFGWG motif. One of the conserved residues in this motif is D381 in VpAAT, whose importance in enzyme activity has been demonstrated by mutagenesis experiments and for other members of the BAHD superfamily. Despite its distance from the active site, the interaction between D381 and Y52 residues is important for maintaining the solvent channel structure in VpAAT [61]. The residue Y52 plays an important role in maintaining enzyme structure (Fig. 2b, c), and the substitution of this residue with phenylalanine leads to a loss in the ability to interact with the substrates. The absence of Tyr significantly reduces (85% reduction) the activity of anthocyanin 5-O-glucoside-6″-O-malonyltransferase [62]. Additionally, the presence of S266 has been shown to be essential for enzyme activity (Fig. 2c). These proteins have an α/β hydrolase fold and a Ser-Asp-His catalytic triad, which is responsible for ester synthesis.

The in silico-modeled AAT reveals the presence of a solvent channel located in between the two domains, which spans the entire protein and allows substrates to reach the catalytic motif at the center of the solvent channel [63]. Although AATs share similar structures and active sites, their substrate specificity varies due to differences in their substrate channels. For instance, a comparison of the protein models of VpAAT and AAT from *Carica papaya* (CpAAT) revealed similar 3D structures for the whole proteins and their catalytic sites. However, CpAAT has a larger solvent channel, which contributes to its higher selectivity for larger acyl-CoA substrates [61, 64]. In a recent study, it was found that four members of AATs from *Cucumis melo* (CmAAT1-4) exhibit differences in their solvent channels [63, 65]. The solvent channel of CmAAT3 is larger, while that of CmAAT1 is smaller, which could partially explain the differences in the biosynthesis of esters by these four AATs. Notably, CmAAT2 does not possess a solvent channel, which leads to failure in ester production [63, 65].

## Active-site and catalytic mechanism of AATs

Despite showing a medium level of sequence identity, AATs exhibit highly conservative 3D structures, with active sites that demonstrate a certain degree of conservation. The active sites of FvAAT are primarily located in two distinct regions, one bound to acyl-CoAs and the other bound to alcohols. These active sites contain a conserved motif HXXXXD, which connects the two regions and forms a substrate reaction channel, enabling the substrate and co-substrate to bind independently. This unique arrangement catalyzes the formation of C–O bonds and facilitates the production of corresponding ester compounds [59, 61, 63].

**Fig. 3:**
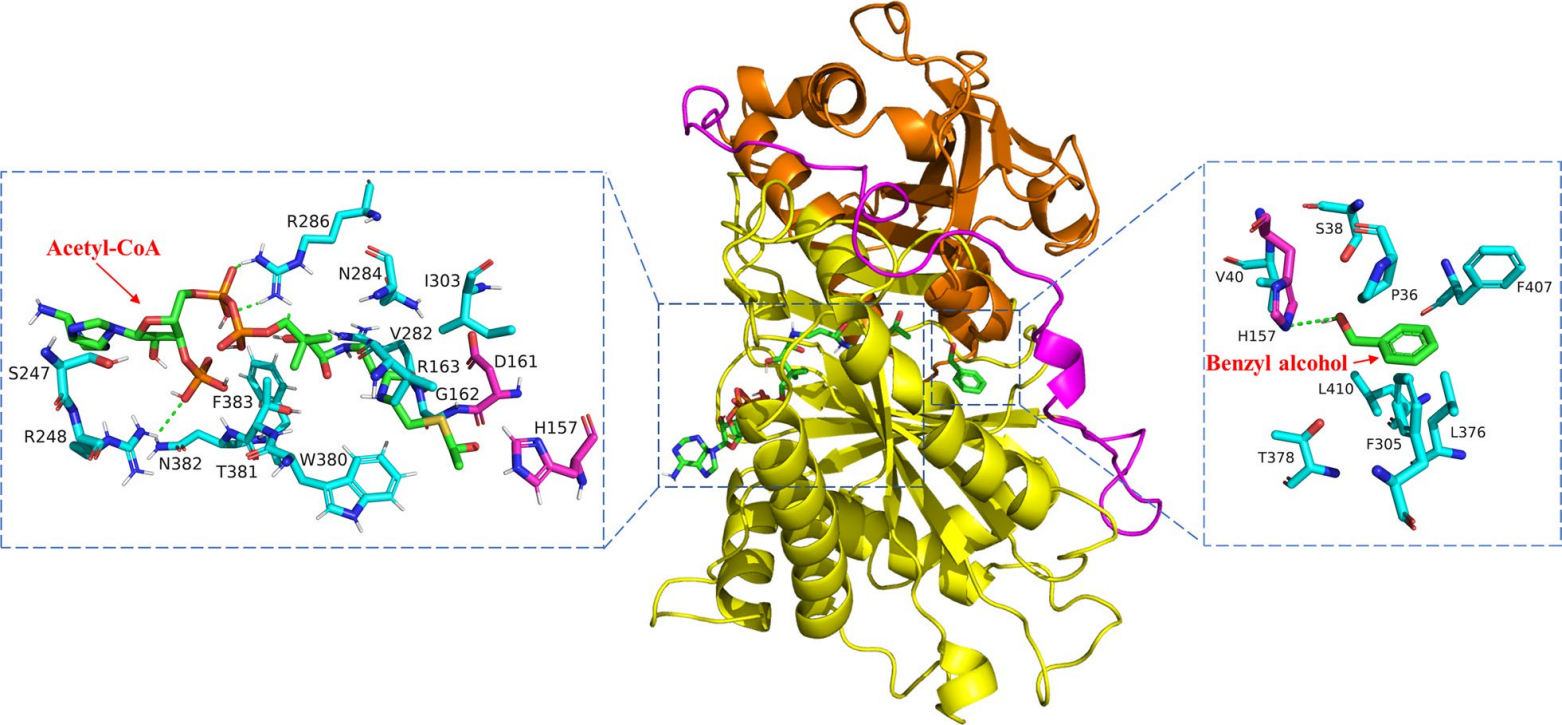
The substrate binding site of FvAAT. The coordinates of benzyl alcohol and acetyl-CoA were manually generated and energetically optimized using the MM2 force field with Chem3D Ultra 8.0. AutoDock (version 3.00) was used for the docking of benzyl alcohol and acetyl-CoA into the homology model of FvAAT by assigning H157 or D161as a grid center. Among the 256 docking poses generated by docking simulations, the one with the minimum docking energy value was selected. Hydrogen bonds are shown with green dotted lines

According to molecular docking results, acetyl-CoA is positioned into the solvent channel entering through the front face and adopting an extended conformation, where the acetyl group is located near the FvAAT active site (Fig. 3). The binding cavity of the FvAAT-acetyl-CoA complex comprises 14 amino acid residues, including S247, R248, N382, T381, F383, W380, R286, N284, V282, R163, G162, I303, D161 and H157. Electrostatic interactions occur between the phosphate groups of acetyl-CoA and positively charged residues R286 and R248 of FvAAT (Fig. 3).

Numerous reports have simulated the dynamic conformation for the binding between acetyl-CoA and AAT. The two positively charged residues R286 and R248 always form hydrogen bonds with the acetyl-CoA phosphate, thereby maintaining acetyl-CoA's position in the active pocket [59, 63, 65]. In FvAAT, the conserved motif HXXXD plays a vital role, with H157 and D161 being crucial for catalytic activity. Mutation of these amino acids to Ala results in a complete loss of catalytic activity. Previous studies have suggested that H166 and D170 of VpAAT in the conserved HXXXD motif played important roles in transferring the acyl group from acyl-CoA to the substrate [60].

Molecular docking studies were conducted to investigate the binding of benzyl alcohol to FvAAT, with the substrate found to be located within the back-face pocket of FvAAT. The binding cavity of the FvAAT–benzyl alcohol complex consists of 14 amino acid residues, including V40, H157, S38, F407, P36, T378, F305, L376, and L410. The hydrophobic amino acid residues in the alcohol binding domain maintain the shape and position of the substrate alcohol binding pocket via van der Waals forces with the substrate, which directly influence the selectivity of the AATs for alcohol substrates [64]. Additionally, the hydroxyl group of the substrate forms hydrogen bond interactions with the Nε atom of residue H157 (Fig. 3), consistent with the docking results of SAAT [66]. The study revealed that the aspartic residue D170 was crucial for maintaining the solvent channel’s structure, whereas H166 was important for the kinetic mechanism [59, 63]. Furthermore, Y52 and D381 play crucial roles in maintaining the stability of the solvent channel [63]. Table 1 summarizes the role of the identified residues in the AAT family proteins, as determined by site-directed mutagenesis studies.

**Table 1:** Residues with important roles identified in the AAT family

Mutants	Sources	Genetic manipulations	References
H166A, D170A, D170N, D170E and S34A	VpAAT	H166A and S34A, D170A and D170N changed the solvent channels in the structure, with no activity for the mutants	[59]
Y52F, D381A and D381E	VpAAT	The binding ability to several substrates was significantly reduced, and D381 and Y52 played a crucial role in maintaining the solvent channel structure	[63]
Y20F, F97W and A138T	CATs	Mutants weaken the interaction between chloramphenicol, H189, and Y20 at the transition state, to improve the promiscuous activities of CATs. Mutants Y20F and F97W improved the promiscuity of CATs towards smaller alcohols and thermostability, but the combinatorial mutations did not result in beneficial effects synergistically	[75, 77, 78]
S405A	CaAT20	The S405A mutant of CaAT increased the volume of enzyme binding cavity, enhanced the affinity with geraniol and thus improved the enzyme activity	[80]
H165V, A170S, A174S, L177T, S262A, F264Y, K298F, F314Y, R339V, K342F, S375A, D376A, H379V, F382Y	PpAAT	Among the 14 candidate amino acid residues, substitutions of S262, F264, and S375 did not cause any significant effect on the enzymatic activities of PpAAT. Substitutions of all 11 candidate amino acid residues possibly involved in the internal esterification reaction dramatically decreased the enzymatic activities of PpAAT, especially the substitutions of H165 and D376, which led to total loss of enzymatic activities	[67]
L41T, F43T, F45T, D169A, R360V, R362V, F372T	PpAAT	Among the 7 candidate amino acid residues, the substitution of F43 did not cause any significant effect on K_*cat*_/K_*m*_ of the site-directed mutant protein. However, substitutions of all the other 6 candidate amino acid residues dramatically decreased K_*cat*_/K_*m*_ of the mutant proteins	[67]
S99G, F185I, L178F	AcAAT	The S99G and L178F mutants produced 4.5-fold and 1.9-fold butyl octanoate compared to WT, respectively, while the L178F mutant produced significantly less butyl butyrate	[72]

AcAAT: AAT from *Actinidia chinensis*

**Fig. 4:**
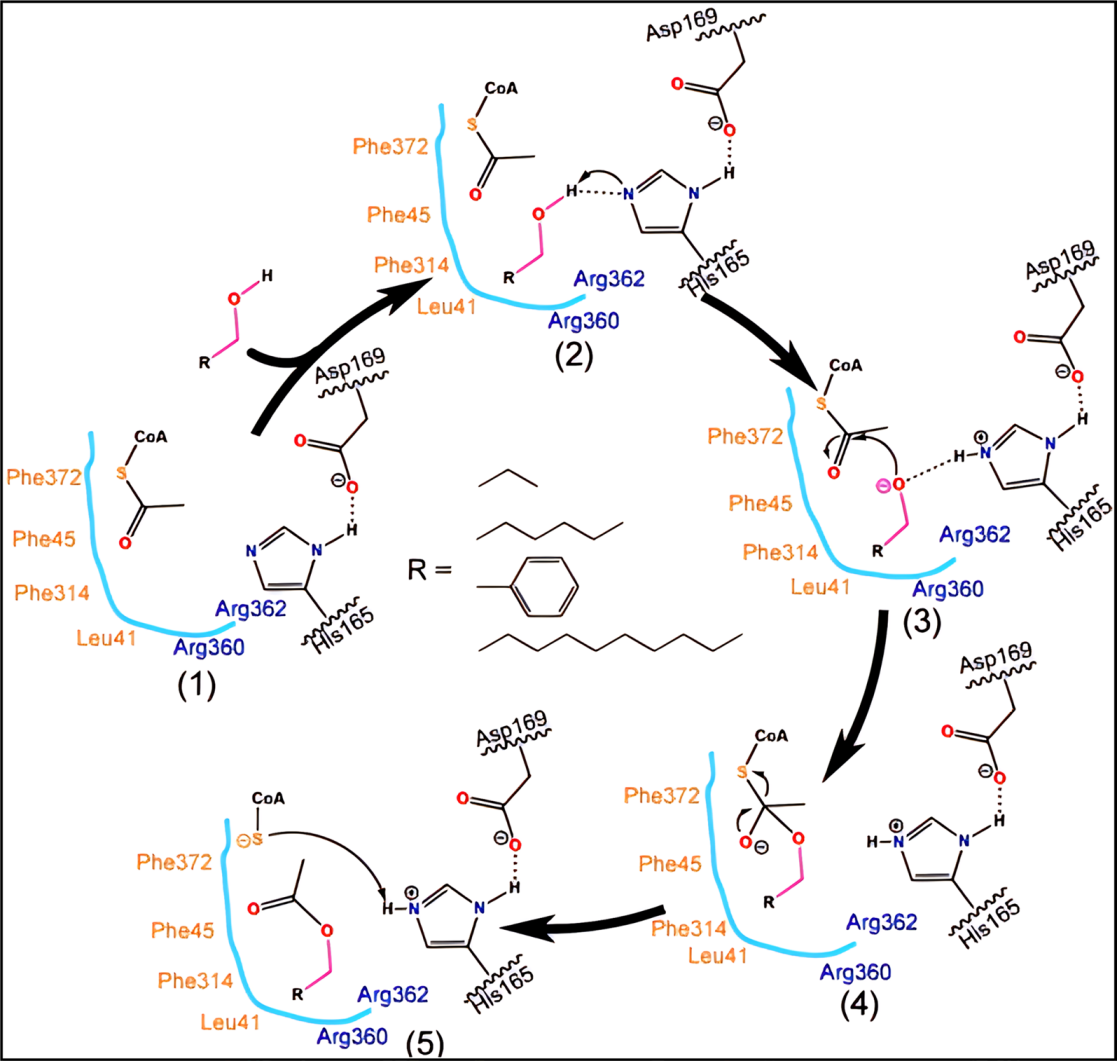
Proposed catalytic mechanism of PpAAT. Esterification reaction using acetyl-CoA and alcohols as substrates. (1) substrates at the catalytic center, (2) the hydroxyl group of alcohols forms a hydrogen bond with His165, (3) a hydrogen atom is transferred to the N atom on His165 and the alcohol substrate forms an O anion to attack the acetyl C atom of acetyl-CoA, (4) the substrates form a C = O double bond and coenzyme A is negatively charged, (5) an ester is formed and the negatively charged coenzyme A accepts a hydrogen atom from His165 to form coenzyme A. This figure is from reference [67]

Song et al. inferred a possible AAT catalytic mechanism by site-directed mutagenesis and molecular dynamics (Fig. 4) [67]. During the esterification reaction of PpAAT, H165 residue directly interact with the substrate, and other amino acid residues participate in substrate recognition and spatial conformation in the reaction center. Initially, H165 is oriented to the carbonyl group of acyl-CoA, and D169 is combined with H165 via hydrogen bond. The alcohol is then redirected to the His residue, leading to the formation of an acyl-CoA-alcohol-His complex. Here, the His residue can deprotonate the hydroxyl group of the alcohol, facilitating its nucleophilic attack on the carbon group of the acyl-CoA to form a C = O double bond and a negatively charged CoA. The ester and the negatively charged CoA receive hydrogen atoms from H165 to form CoA. Finally, the acyl-CoA-SH group is removed, resulting in the formation of the corresponding ester products. The proposed AAT catalytic mechanism involves the creation of a ternary complex among the acyl-CoA, alcohol and protein. This mechanism is consistent with the acyl transfer mechanistic features described for other BAHD family members such as 5-*O*-glucoside-6″-*O*-malonyltransferase from *Salvia splendens* flowers (Ss5MaT1), chloramphenicol *O*-acetyltransferase (CAT), and histone acetyltransferase (HAT), which have been extensively studied [68–71].

## Protein engineering of AATs

The application of AAT as a key enzyme for the synthesis of ester compounds is fraught with challenges such as unsatisfactory catalytic activity and selectivity, poor thermal stability, and low solubility of heterologous expression. Many researchers have tried to improve the properties of AATs by protein engineering and achieved some success.

### Increasing the activity of AATs towards acyl-CoAs

In the catalytic mechanism of AATs, the H residue of motif HXXXD can deprotonate the hydroxyl group of the alcohol, facilitating its nucleophilic attack on the carbonyl group of acyl-CoAs [59, 63]. Molecular dynamics simulations have shown that the distance between the acyl-CoA substrate and the His residue remains relatively unchanged throughout catalysis [59, 63]. Micaela et al. hypothesized that certain amino acid mutations might alter the orientation of the octanoyl-CoA substrate in the solvent channel, improving its position relative to H166 [72]. Further analysis revealed that the unfavorable distance (9.7 Å) between the octanoyl-CoA substrate and H166 was the reason for the low activity of AAT16 in producing octanoyl esters. Two mutants (S99G and L178F) were obtained by substrate docking analysis, which reduced the distance between octanoyl-CoA and H166 to 7.97 Å and 8.8 Å, respectively [72]. Experimental investigations confirmed that the AAT16-S99G and AAT16-L178F mutants produced 4.5-fold and 1.9-fold more butyl octanoate than AAT16-WT. However, the double mutant AAT16-S99G-L178F did not further improve the titer, and the composition of the produced ester was similar to the AAT16-S99G single mutant. Additionally, an F185I mutant, which increased the distance (12.7 Å) between octanoyl-CoA and the catalytic base (in silico) was selected for comparison. As expected, the AAT16-F185I mutant had significantly reduced ester production compared to AAT16-WT [72]. This method provides a promising idea for AAT engineering, especially for improving the catalytic activity and substrate specificity of acyl-CoAs. The catalytic activity and selectivity of the mutant to the substrate acyl-CoAs were determined by substrate docking analysis using the distance between acyl-CoAs and the His residue.

### Increasing the activity of AATs towards alcohols

Furthermore, there have been efforts to improve the affinity of AATs to alcohols through protein engineering. For instance, wax ester synthase/acyl-coenzyme A: diacylglycerol acyltransferase (WS/DGAT), a member of AATs family, utilizes fatty alcohols and fatty acyl-CoAs to synthesize the corresponding wax esters. WS/DGAT from the *Marinobacter aquaeolei* VT8 (Mal) have the most favored substrates with undecanol and dodecanol [73]. Barney et al. changed substrate selectivity of Mal for alcohol by introducing amino acid residues with larger volume into the active pocket. The mutant Ma1-A360I has higher selectivity to decanol and undecanol, and significant increases in selectivity were found for several smaller fatty alcohols [73]. Valle‑Rodríguez et al. implemented a random mutagenesis approach to enhance the catalytic efficiency of a WS from *Marinobacter hydrocarbonoclasticus* (MhWS2). The mutant MhWS2-A344T substantially increases the selectivity toward ethanol and other shorter alcohols, due to change of both steric effects and polarity changes near the active site [74]. Seo and Lee et al. demonstrated that the F97W mutant of CAT from *Staphylococcus aureus* (CATsa-F97W) improved the activity towards isobutanol [75, 76] and Kobayashi et al. demonstrated that CATsa-A138T increased thermostability [77]. Seo et al. found that CATsa-Y20F improved the catalytic efficiency over CATsa and CATsa-F97W by 5.0- and 2.5-fold, respectively, while the melting temperature was slightly decreased from 71.2 to 69.3 ℃ [78]. Among the combinatorial mutagenesis, CATsa-Y20F-A138T exhibited the highest melting temperature (76 ℃). Unfortunately, the combinatorial mutation of F97W and Y20F did not further improve the catalytic efficiency and lowered the melting temperature. The combinatorial mutant CATsa-Y20F-A138T-F97W not only reduced the activity towards isobutanol, but also lowered the melting temperature [78]. Seo et al. also screened CAT from *Clostridium thermocellum* (CATec3) which could retain more than 95% of the activity at 70 ℃ while the residual activity of CATsa rapidly decreased. Interestingly, the mutant CATec3-Y20F improved not only the catalytic efficiency, ~ 3.3-fold higher than WT, but also the melting temperature (87.5 ℃) [78]. The mutant CATsa-Y20F did not improved thermostability, suggesting that the effect of Y20F on thermostability might vary among different CATs. Seo et al. successfully engineered chloramphenicol acetyltransferases (CATs) from mesophilic prokaryotes to function as robust and efficient AATs compatible with at least 21 alcohol and 8 acyl-CoA substrates for microbial biosynthesis of linear, branched, saturated, unsaturated, and/or aromatic esters [78]. This example also provides a unique direction to increase the thermal stability of AATs, which can increase the volatile collection rate of esters from aqueous solution during fermentation and reduce the toxicity of high-concentration esters to cells, leading to increased production of esters. CATec3-Y20F, a mutant with higher solubility, thermostability, and selectivity, has been utilized for designer acetate ester production [79]. Reshaping the active pocket of AATs has also been successful in increasing catalytic activity. For example, Yan et al. selected S405 located substrate binding region in AAT from *Celastrus angulatus* Maxim (CaAT20) as “hot spots” for mutation analysis and obtained mutant S405A could increase the catalytic activity for geranyl benzoate [80]. Molecular simulation results showed that the binding cavity volume of S405A mutant was much larger than that of WT.

Combinations of beneficial single-site mutations have yielded cumulative or synergistic effects on thermal stability and enzymatic activity. For example, Le et al. screened and analyzed the potential important mutation sites of AAT from *Wickerhamomyces anomalus* YF1503 (Eat276) with the help of HotSpot Wizard and FireProt [81]. The obtained mutant Eat276-M89P significantly increased the enzyme activity (~ 2-folds higher than WT) and the mutant Eat276-R261L significantly increased the thermal stability (6.2 ℃ higher than WT). In addition, both Eat276-F161M and Eat276-F161L increased the enzymatic activity [81]. Then, the authors combined these single-site mutations and obtained the combinatorial mutant Eat276-R261L-M89P-F161L, whose melting temperature and specific enzyme activity was increased by 10 ℃ and ~ 4.9-fold, respectively [81].

### Increasing the soluble expression of AATs

**Fig. 5:**
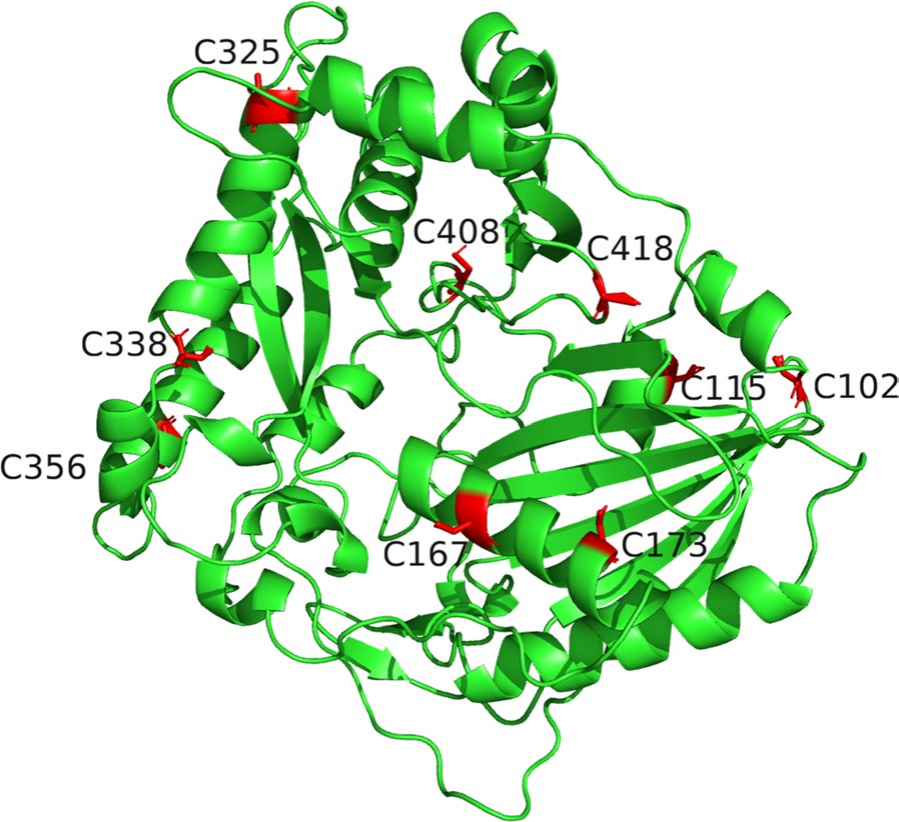
Distribution of Cys residues on the surface of FvAAT. FvAAT is shows green cartoon, and Cys residues of FvAAT are represented as sticks colored in red

A majority of AATs are derived from plants, and heterologous expression in microorganisms is challenging. One significant issue is the low solubility of eukaryotic AATs in microbial hosts [82, 83]. Strategies such as codon optimization, fusion partners, and co-expression of molecular chaperones have been employed to improve the soluble expression of AATs [84]. Based on the structure of AATs, there are numerous Cys residues on the surface of AATs, limiting the solubility of AATs. For instance, there are ten Cys residues on the surface of FvAAT (Fig. 5). Previous studies have found that replacing the surface Cys residues could significantly increase the soluble expression of heterologous proteins. To improve the soluble expression and whole-cell catalytic activity of AAT in *Escherichia coli*, Fujio et al. performed alanine mutations on the surface amino acids of AATs from various sources (e.g., apple, tomato, and strawberry) [85]. Our laboratory also confirmed this strategy's effectiveness by mutating the Cys residues on the surface of FvAAT. However, there are limited examples of protein engineering modifications of AATs, due to a lack of crystal structure data available for AATs. Moreover, homology modeling is only possible using other members of the BADH family as templates. Soluble expression of AATs is essential for obtaining AAT proteins for protein structural studies, enabling deeper analysis of catalytic mechanisms and more favorable conditions for rational protein engineering.

## AATs-mediated metabolic engineering for ester production

**Fig. 6:**
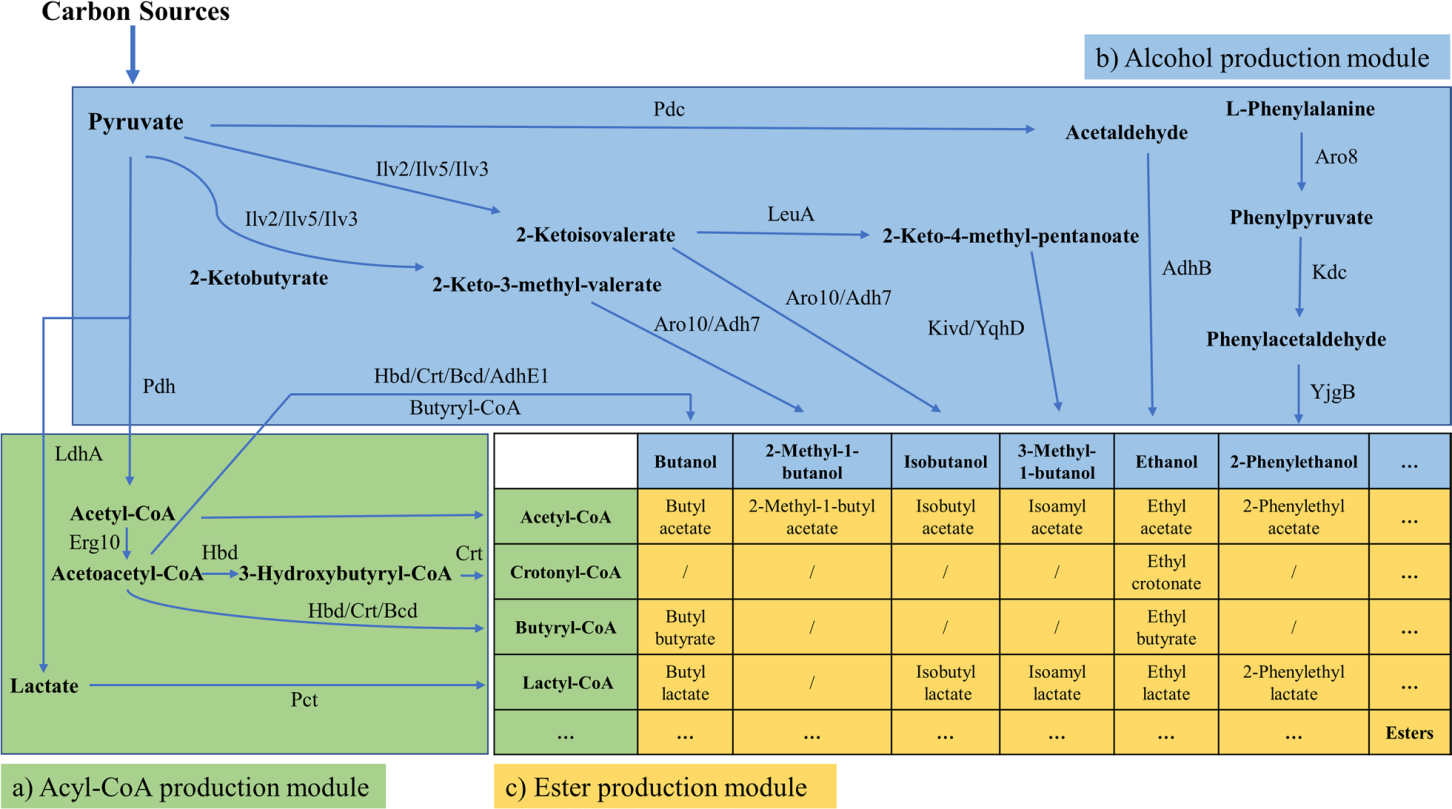
Overview of the metabolic pathways for microbial biosynthesis of esters. **a** Acyl-CoA production module. Acyl-CoAs are mainly synthesized with acetyl-CoA as the precursor. **b** Alcohol production module. Alcohols are formed via the keto acid pathway. **c** Esters production module. AATs catalyze the condensation of acyl-CoAs and alcohols to form the corresponding esters. Pdh, pyruvate dehydrogenase; IdhA, lactate dehydrogenase; Erg10, acetyl-CoA acetyltransferase; Hbd, 3-hydroxybutyryl-CoA dehydrogenase; Crt, 3-hydroxybutyryl-CoA dehydratase; Pct, propionate CoA-transferase; Pdc, Pyruvate decarboxylase; Adh, alcohol dehydrogenase; Ilv2, acetolactate synthase; Ilv5, acetohydroxyacid reductoisomerase; Ilv3, dihydroxyacid dehydratase; Aro10, ketoacid decarboxylase; LeuA, 2-isopropylmalate synthase; Kivd, 2-ketoisovalerate decarboxylase; Bcd, butyryl-CoA dehydrogenase; YqhD, alcohol dehydrogenase; Aro8, aminotransferase; Kdc, ketoacid decarboxylase; YjgB, NADPH-dependent aldehyde reductase; AAT, alcohol acyltransferase

The microbial biosynthesis of esters involves three primary modules: the acyl-CoA production module, the alcohol production module, and the ester biosynthesis module [86] (Fig. 6). The ester biosynthesis module largely depends on the selection of appropriate AAT enzymes [87]. Acetic esters are currently the most commonly synthesized esters compounds by microorganisms because the precursor, acetyl-CoA, is naturally abundant in all organisms without the need for exogenous gene introduction [88]. In addition, there have been different strategies to enhance the production of acetyl-CoA. For instance, by removing competing pathways (Δ*ldh*, Δ*poxB*, Δ*pta*) and overexpressing AAT, 36 g/L isobutyl acetate was obtained in a 1.3 L bench-scale bioreactor [82]. Kong et al. reported the synthesis of indole-3-ethanol acetate by directly overexpressing AAT from renewable carbon sources [89]. Shi et al. impeded mitochondrial transport and pyruvate and acetyl-CoA consumption to increase ethyl acetate accumulation in the cytoplasm [90]. Additionally, complex esters (such as ethyl crotonate [91], isobutyrate, butyrate [92], lactate [93], etc.) can be synthesized by introducing uncommon acyl-CoA biosynthetic pathways. Zhang et al. obtained crotonyl-CoA by introducing genes *Erg*, *Hbd* and *Crt* into *Saccharomyces cerevisiae*, and then catalyzed the synthesis of ethyl crotonate by AAT in the presence of another substrate ethanol [91]. Sato et al. treated methacrylyl-CoA with an alcohol in the presence of AAT to synthesize methacrylic acid ester [94]. Kenji et al. produced 3-hydroxyisobutyric acid ester with alcohol and 3-hydroxyisobutyryl-CoA in the presence of AAT [95]. Chen et al. produced ethyl hexanoate, ethyl octanoate, and ethyl decanoate by heterologously overexpressing the alcohol acyltransferase gene [96]. Furthermore, the supplementation of acids can increase the availability of acyl-CoAs [97]. Similar strategies can also increase the supply of alcohols. Strategies to enhance precursor supply have been previously reviewed and will not be redundantly described in detail here [49].

In the last module, the selection of an appropriate AAT plays a crucial role in determining the final ester products. AATs possess a wide range of substrate specificity and can recognize various acyl-CoA molecules, including acetyl-CoA, propionyl-CoA, butyryl-CoA, and others. The specificity of the acyl-CoA substrate determines the type of ester produced, such as acetate esters [90], propionate esters [87], lactate esters [93], butyrate esters [98, 99], pentanoate esters [87], hexanoate esters [98] and so on.

AATs also demonstrate a wide range of specificity for other substrate alcohols, including ethanol, butanol, isoamyl alcohol, benzyl alcohol, geraniol and others. Similarly, the type of alcohol substrate also affects the type of product esters. Thus, the selection of alcohols and acyl-CoAs ultimately determines the production of a specific ester with a suitable AAT. Furthermore, the versatility and broad substrate specificity of AATs permit the artificial design of novel esters that may not exist naturally.

Considering the significant role of AATs in the design of a metabolic pathway for ester synthesis, Zhang et al. screened several AATs, including SAAT, AAT from *Fragaria chiloensis* (FcAAT), AAT from *Vasconcellea cundinamarcensis* (VcAAT), FvAAT, AAT from *Vitis* × *labruscana* (VlAAT), and AAT from *Pyrus ussuriensis* (NgAAT), and identified SAAT as an effective catalyst for the biosynthesis of ethyl crotonate [91]. Similarly, Lee et al. screened SAAT and VAAT to synthesize lactate esters [93]. Rapid screening of suitable AATs required for ester biosynthesis brings convenience to ester biosynthesis design. Lee et al. developed a high-throughput microbial screening platform for AATs by combining microplate-based culturing techniques with a colorimetric assay [76]. This platform could not only probe the alcohol substrate specificity of both native and engineered AATs, but also identify the beneficial mutations in engineered AATs for enhanced ester synthesis. Zhang et al. summarized recent development on the biosynthesis of alkyl esters and focused mainly on the enzyme engineering strategies of critical wax ester synthases, and the pathway engineering strategies employed for the biosynthesis of various fatty acid alkyl ester products [22]. More metabolic engineering examples of ester biosynthesis are summarized in Table 2.

**Table 2:** Overview of metabolic engineering for microbial ester production

Enzyme source	Production host	Ester products	Titer	Genetic manipulations	References
*S. cerevisiae* *(ATF1)*	*Clostridium autoethanogenum*	Ethyl acetate	0.3 mM	Using CO-based feedstocks for microbial ester production	[104]
		Butyl acetate	4.5 mM		
*F. ananassa* *(AAT)*	*S. cerevisiae*	Ethyl crotonate	125.59 ± 2.04 mg/L	The expression cassettes with different strengths to regulate the expression of key genes	[91]
*S. cerevisiae (ATF1)*	*E. coli*	Neryl acetate	11.712 ± 0.653 mg/L	Overexpression of tHMG1 and supplementation with 2 g/L pyruvate	[105]
*S. cerevisiae (ATF1)*	*E. coli*	Isobutyl acetate	2.48 g/L	Removing byproduct pathways (*Δldh, ΔpoxB, Δpta*) and overexpressing AAT	[82]
*S. cerevisiae (ATF1)*	*E. coli*	Isoamyl acetate	0.78 g/L		
*Staphylococcus aureus* *(CAT)*	*E. coli*	Isoamyl acetate	8.8 g/L	A systematic modular design approach to control proteome reallocation for the selective microbial biosynthesis of branched-chain acetate esters via manipulation of substrate specificity and expression level of multiple pathway enzymes	[79]
*S. cerevisiae (ATF1)*	*Pseudomonas putida KT2440*	Hexyl acetate	160.5 mg/L	Overexpression of transporters	[106]
*V. cerevisiae* *(ATF1)*	*E. coli*	Indole-3-ethanol acetate	550 ± 24.05 mg/L	Biosynthesis of indole-3-ethanol acetate directly from a renewable carbon source	[89]
*S. cerevisiae (ATF2)*	*industrial yellow rice wine yeast strain*	Ethyl acetate	137.79 mg/L	Overexpressing the alcohol acetyltransferase-encoding gene ATF2	[107]
		Isoamyl acetate	26.68 mg/L		
		Isobutyl acetate	7.60 mg/L		
*Wickerhamomyces anomalus* *(EAT1)*	*S. cerevisiae*	Ethyl acetate	6.48 ± 0.32 g/L	A novel alcohol acetyltransferase family	[42]
*S. cerevisiae (ATF1)*	*E. coli*	Isobutyl acetate	17.2 g/L	Engineered *E. coli* has an ability to produce acetate ester, isobutyrate ester, butyrate ester	[7]
		Tetradecyl acetate	137 mg/L		
*Strawberry* *(AAT)*	*S. cerevisiae*	Ethyl hexanoate	42.35 ± 1.75 mg/L	Optimizing the synthetic pathway of ethyl hexanoate	[108]
*S. cerevisiae (ATF1)*	*E. coli*	Farnesyl acetate	128 ± 10.5 mg/L	Biosynthesis of the advanced biofuel farnesyl acetate directly from glucose	[109]
		Farnesyl acetate	201 ± 11.7 mg/L		
*Strawberry* *(AAT)*	*S. cerevisiae*	Ethyl butyrate	99.65 ± 7.32 mg/L	Introduced a butyryl-CoA synthesis pathway into *S. cerevisiae*	[110]
*Kiwifruit* *(AeAT9)*	*S. cerevisiae*	Ethyl acetate	1.69 g/L	Impeded mitochondrial transport and utilization of pyruvate and acetyl-CoA to increase the ethyl acetate accumulation in the cytoplasm	[90]
*Strawberry* *(AAT)*	*Clostridium acetobutylicum*	Butyl butyrate	40.60 mg/L	The one-step fermentation of butyl butyrate from glucose in the engineered *C. acetobutylicum*	[99]
*Apple* *(AAT)*			50.07 mg/L		
*S. cerevisiae (ATF1)*	*E. coli*	2-Phenylethyl acetate	687 mg/L	An economical process for the biosynthesis of 2-PEAc directly from glucose	[111]
*S. cerevisiae (ATF1)*	*S. cerevisiae*	Isobutyl acetate	260.2 mg/L	Increase the ester production by improving the mitochondrial pyruvate concentration towards branched-chain alcohol biosynthesis	[112]
		3-Methyl-1-butyl acetate	296.1 mg/L		
		2-Methyl-1-butyl acetate	289.6 mg/L		
*Rosa hybrida* *(AAT)*	*E. coli*	Geranyl acetate	10.36 g/L	Biosynthesis of monoterpene esters	[113]
*Strawberry* *(AAT)*	*E. coli*	Butyl acetate	0.64 g/L	Engineer modular microbial platforms for anaerobic production of butyryl-CoA-derived designed esters from renewable feedstocks	[84]
		Butyl butyrate	0.45 g/L		
		Ethyl butyrate	0.41 g/L		
*S. cerevisiae (ATF1)*	*E. coli*	Anisyl acetate	355 mg/L	An efficient artificial biosynthetic pathway from glucose to anisyl acetate	[114]
*S. cerevisiae(ATF1)*	*E. coli*	Butyl acetate	22.8 g/L	Optimization of AAT expression and redox balance with auto-inducible fermentative controlled gene expression	[115]
*Marinobacter hydrocarbonoclasticus* *(WS2)*	*S. cerevisiae*	Fatty acid ethyl ester	5 g/L	Push–pull-block strategy	[116]
*S. cerevisiae (ATF1)*	*E. coli*	Isoprenyl acetate	28 g/L	Harnessing the IPP-bypass MVA pathway	[117]
*Strawberry* *(AAT)*	*Clostridium tyrobutyricum*	Butyl butyrate	62.59 g/L	Under mannitol with fed-batch fermentation in a 5 L bioreactor, which is the highest butyl butyrate titer reported so far	[118]

## Challenges and perspectives

This review provides a comprehensive overview of AATs, including sequences, structures, catalytic mechanisms, and metabolic engineering applications. As the important enzymes in ester biosynthesis, AATs play a vital role in determining the final ester product. Although some progress on AATs has been made, it still faces several grand challenges for further engineering of ester biosynthesis.

The first major challenge is the expansion of the AAT enzyme library. Due to the different substrate specificity of AATs, it is necessary to pair specific AAT to meet the efficient synthesis of different esters. Currently, the number of identified AATs is still rather limited, and the substrate spectrum of most identified AATs remains fully uncharacterized, which limit their applications in biosynthesis of various esters. Therefore, mining unknown AATs and exploring their substrate spectrum is crucial for designing the ester biosynthetic pathways. In addition, protein engineering offers a powerful tool to address challenges in low catalytic activity and poor substrate specificity of AATs for broadening biotechnological applications [78]. While the testing of AAT mutants can be labor-intensive and time-consuming, advancements in automated facilities (e.g., BioFoundry [100]) and high-throughput screening [76] are expected to facilitate the engineering of AATs with the desired enzymatic properties. What is more, AI-driven protein design [101] provides another paradigm in AAT engineering.

The second challenge is to study the catalytic mechanism of AATs. Unfortunately, no crystal structure data of AAT are available in the PDB database, and enzymes of other BADH families are chosen as templates for protein homology modeling. Although alpha-fold 2 has produced a more accurate model, the lack of precise end data limits the catalytic mechanism study of AAT's. The lack of structural data on AAT may be due to limited research on its application. Over the past decade, the importance of ester synthesis has become a key component for the development of renewable resources, and research on its mechanism has gradually increased. Recently, alphaFold, which uses deep neural networks to predict the folding of a protein based on its amino acid sequence [102], has the potential to revolutionize protein researches, including the understanding of the catalytic mechanisms of AATs. The accurate AAT models generated by alphaFold enable the elucidation of the structure and function relationship, which helps us to identify and engineer the needed AATs.

The third challenge lies in their molecular modification. While AATs have a wide range of substrate specificity and can synthesize various types of esters, it exhibits low activity for the synthesis of certain specific esters, such as methyl lactate. Molecular modification can be employed to modify its active pocket and substrate channel to enhance catalytic efficiency. Additionally, several natural and non-natural esters have not been successfully synthesized with AATs yet. The broad substrate selectivity of AATs is both advantageous and disadvantageous. While it facilitates the exploration of new functions of AATs, it also results in the production of many by-products during ester biosynthesis, resulting in higher non-target ester yields than target esters [82, 90, 98]. For instance, the efficiency of engineering microorganisms to synthesize butyrate esters is much lower than that of acetate esters [103]. An engineered *E. coli* with a butyrate ester pathway can generate two acyl-CoAs (acetyl-CoA and butyryl-CoA) and two alcohols (ethanol and butanol), forming four possible esters (ethyl acetate, butyl acetate, ethyl butyrate, and butyl butyrate). In the case of the lactate ester synthesis pathway constructed in engineered *E. coli*, the yield of the ethyl lactate (1.59 mg/L) was much lower than that of ethyl acetate (115.52 mg/L) [92]. The formation of these by-products not only decreases product yield but also results in more complicated steps and higher costs for subsequent separation and purification. Therefore, efficient AATs with high substrate specificity are critical for high-level microbial biosynthesis of a target designed ester [78]. While numerous metabolic platforms have been established in cell factories for ester synthesis, further enhancements in productivity are necessary to achieve industrial-scale production and compete with traditional petrochemical counterparts. The cooperative action of enzyme engineering and metabolic pathway engineering is crucial for fermentative production of tailored ester compounds with high selectivity and efficiency.

## Conclusions

With the emergence of synthetic biology tools, it has become possible to synthesize various esters from renewable sources, such as biomass and even carbon dioxide, thereby reducing the dependence on petrochemical energy. However, the low efficiency of AATs poses the biggest challenge for the biosynthesis of esters, making it costly and inefficient for industrial production. This review summarizes the sequences, structures, and catalytic mechanisms of AATs, and provides perspectives on the design of more efficient ester biosynthetic pathways. With challenges on ester biosynthesis, further engineering of AATs should be performed to construct microbial cell factories for the efficient biosynthesis of esters.

## Data Availability

Not applicable.
